# Chitosan as a Plurivalent Biopolymer in Nanodelivery Systems

**DOI:** 10.3390/polym17050558

**Published:** 2025-02-20

**Authors:** Marius Gabriel Dabija, Iulia Olaru, Tudor Ciuhodaru, Alina Stefanache, Cozmin Mihai, Ionut Iulian Lungu, Gabriela Calin, Carmen Stadoleanu, Daniela Liliana Damir

**Affiliations:** 1 “Grigore T. Popa” University of Medicine and Pharmacy, 700115 Iasi, Romania; 2 Faculty of Medicine and Pharmacy, “Dunarea de Jos” University, 47 Domneasca Str., 800008 Galati, Romania; 3 Faculty of Dental Medicine, “Apollonia” University of Iasi, 11 Pacurari Str., 700511 Iasi, Romania

**Keywords:** biopolymers, chitosan, nanodelivery systems, nano-architecture

## Abstract

(1) Background: This review study will delve into the potential of chitosan nanoparticles (NPs) as adaptable carriers for targeted drug delivery in different therapeutic areas. Chitosan is a biopolymer derived from chitin that has attracted interest in drug delivery applications because of its high biocompatibility and biodegradability. (2) Methods: A comprehensive literature review was conducted by following a careful systematized protocol for searching databases like PubMed, Google Scholar and ScienceDirect. (3) Results: Chitosan NPs are good drug delivery vehicles, notably for cancer. Studies reveal that doxorubicin-loaded chitosan NPs dramatically enhance toxicity to tumor cells compared to free medicines, yielding tumor suppression rates of up to 60%. Researchers found that chemotherapeutics had an 85% encapsulation efficiency (EE), lowering systemic toxicity. Magnetic and pH-responsive chitosan NPs boost drug accumulation by 63% and apoptosis by 54%. Chitosan also boosts medication retention in the lungs by 2.3×, per pulmonary delivery trials. Chitosan NPs also boost ocular medication bioavailability by 3× and improve nasal absorption by 30%, crossing the blood–brain barrier. For bone regeneration, chitosan scaffolds enhance bone mineral density by 46%, facilitating osteogenesis and healing. (4) Conclusions: NPs made of chitosan provide a solid foundation for improving drug delivery systems; yet there are still issues with material variability, scalability, and meeting regulatory requirements that need fixing. Research into combination treatments, ways to increase their specificity, and ways to optimize these NPs offers promising prospects for the creation of novel therapeutic approaches with the potential to improve patient outcomes.

## 1. Introduction

**Figure 1:**
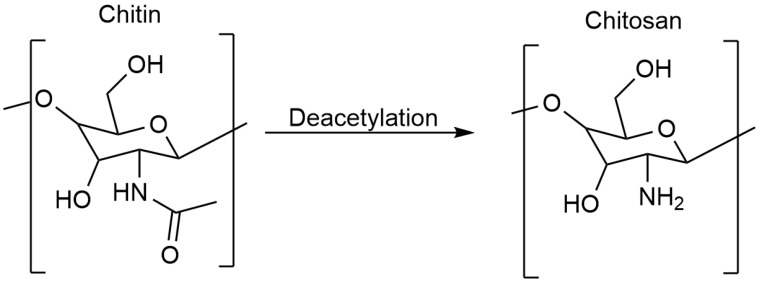
The deacetylation reaction for transforming chitin into chitosan.

Chitosan is a biopolymer made from chitin; it is mostly created by deacetylating chitin (Figure 1), a substance that is prevalent in the exoskeletons of crustaceans like crabs and shrimp. Chitosan has certain physicochemical features that make it useful in biomedical applications, thanks to its linear chain of β-(1→4)-linked N-acetyl-D-glucosamine and D-glucosamine units. The cationic properties imparted by the amino and hydroxyl groups within chitosan’s structure make it soluble in acidic environments [1,2,3]. NPs formed from this soluble chitosan can carry different drug molecules, improving the efficacy of drug delivery across biological barriers. With the increasing need for safer medicinal products, chitosan’s outstanding biocompatibility and biodegradability make it an attractive alternative to conventional drug delivery systems, which frequently suffer from issues like systemic toxicity, poor solubility, and quick degradation. At the same time, chitosan NPs provide a workable alternative as they enable the encapsulation of hydrophobic and hydrophilic medications and encourage the regulated and prolonged release of medicinal compounds [4,5,6,7]. According to Jin et al. (2014), chitosan NPs may increase the bioavailability of paclitaxel and other medicines that are not very water-soluble [8].

Chitosan is well-known for its bioadhesive capabilities, which allow NP formations to remain at the site of action for an extended period of time, improving drug absorption, in addition to its excellent physicochemical features. Applications involving mucosal surfaces, such as ophthalmic, nasal, and gastrointestinal drug delivery systems, greatly benefit from this mucoadhesion. Animal studies have shown that chitosan NPs had better therapeutic outcomes than conventional formulations due to their increased adhesion to mucosal surfaces [9,10,11,12].

Extensive research on the potential uses of chitosan NPs in cancer, gene therapy, and antibacterial treatments has been conducted. By adjusting chitosan NPs’ surface properties, scientists may create tailored drug delivery systems that deliver medications to certain cells or tissues. One example is the work of Maeda and Kimura (2004), who demonstrated the immunomodulatory and direct drug delivery capabilities of low-molecular-weight chitosan by demonstrating their ability to activate natural killer (NK) cells in tumor-bearing mice [13].

Multifunctional chitosan-based nanocarriers that can react to particular stimuli, including pH changes or enzyme activity, have been developed thanks to ongoing breakthroughs in nanotechnology and material science. By releasing therapeutic molecules at the precise moment and place needed, this stimulus-responsive behavior has the potential to radically alter medication delivery, increasing efficiency while decreasing adverse effects [14,15,16,17].

The use of chitosan, a plurivalent biopolymer, in nanodelivery systems is becoming more apparent as nanomedicine develops further. Current research results, mechanisms of action, practical consequences, and future research routes targeted at maximizing their usage in therapeutic settings are examined in this article, which also delves into the broad potential of chitosan NPs in drug delivery applications. Chitosan is an attractive contender for tackling some of the most critical problems in medication delivery due to its adaptability, natural origin, and beneficial characteristics [18,19,20]. Researchers want to improve patient outcomes and lay the groundwork for next-gen targeted and personalized medicine by capitalizing on its unique traits to build revolutionary treatment tactics.

## 2. Drug Delivery Mechanisms and Applications

Passive diffusion and active transport are the two main ways in which chitosan NPs help in medication delivery. Therapeutic medicines may accumulate within specific cells by passive diffusion, which enables pharmacological substances to cross cell membranes propelled by concentration gradients. The formulation and the drug’s physicochemical qualities are crucial to this procedure [21,22,23,24].

On the other hand, cellular absorption is enhanced by active transport processes when negatively charged components of cell membranes, such as phospholipids and glycoproteins, interact with the cationic surface of chitosan. For medications, especially hydrophilic ones, to be better absorbed by cells, this interaction is essential. Environmental pH and ionic strength are two of the changeable elements that impact the efficacy of these pathways and may greatly regulate release rates. One example is the fundamental impact that chitosan NPs’ pH sensitivity has in their drug release profile [25,26,27,28,29]. Chitosan NPs have mucoadhesive characteristics that increase the amount of time they spend in touch with epithelial surfaces in the intestines, according to research by Shi et al. (2002), which emphasizes the significance of these environmental variables. Improved medication absorption, a key component of increasing bioavailability, may result from this longer contact [1].

The results of quantitative investigations on the sensitivity of chitosan NPs to changes in pH in the environment are very encouraging. Chitosan NPs enlarge substantially when the pH is set to about 5.5, which is characteristic of certain pathological settings like tumor sites or irritated tissues. The swelling effect improves the release of medications that are encapsulated [30,31,32,33,34].

Chitosan NPs have a double purpose: they may be used for medication delivery and to create controlled-release formulations that are customized to a patient’s needs, so that the medicine is as effective as possible while causing as few adverse effects as possible [14,35,36,37].

### 2.1. Application in Cancer Therapy

With great promise for better treatment results in cancer, chitosan NPs have arisen as strong contenders for the targeted delivery of chemotherapy drugs. Anticancer medications may be optimally encapsulated and released by chitosan because of its inherent features, such as its biodegradability, capacity to create stable NPs, and biocompatibility.

Research suggests that systems based on chitosan may improve the bioavailability and therapeutic effectiveness of chemotherapy by changing the tumor microenvironment. As an example, Cheng et al. (2009) created doxorubicin-loaded chitosan NPs and showed that these formulations were far more hazardous to several tumor cell types than free doxorubicin [38]. In vivo trials demonstrated tumor suppression rates of up to 60%, highlighting the potential of chitosan NPs to reduce systemic exposure and related adverse effects while delivering payloads specifically to tumor locations [39,40,41].

Besides their potential use in local distribution, chitosan NPs have characteristics that might affect immune regulation in cancer treatment. Mice with malignancies may have their NK cells stimulated by low-molecular-weight chitosan, according to research by Maeda and Kimura (2004). Based on these findings, chitosan NPs might have dual roles: delivering chemotherapeutic drugs and boosting the host immune response to malignancies. These formulations have the ability to improve the efficacy of immunotherapeutic techniques when used in conjunction with traditional chemotherapies by increasing NK cell activation [13]. To ensure that medications are delivered exactly where they are required, chitosan NPs may be programmed to release their contents in response to specific triggers inside the tumor microenvironment, such as acidity or high levels of certain enzymes.

The high EE, controlled drug release, and tumor-targeting features of chitosan-based NP (NP) delivery systems make them very promising for cancer treatment. The mucoadhesive and biocompatible properties of chitosan were highlighted by Baharlouei and Rahman (2022), who reported an EE of more than 85% for a range of chemotherapeutics, resulting in better drug stability and less systemic toxicity [42]. Drug-loading capabilities (DLCs) of up to 40%, approximately double that of typical polymeric systems, are highlighted in additional detail by Tian et al. (2023) for multi-functional chitosan NPs [43]. A meta-analysis by Ahmad et al. (2022) demonstrates that chitosan NPs increase bioavailability and prolong therapeutic effects by 2.5 to 3 times, as they prolong the half-life of drugs [44]. Optimizing drug delivery while avoiding side effects, Rajaei et al. (2023) show that a chitosan/agarose/graphene oxide nanohydrogel produces a sustained 5-fluorouracil release of 78% over 72 h, compared to just 45% in non-chitosan formulations [45].

Magnetic and pH-responsive chitosan-based technologies further improve targeted medication delivery. Compared to free medicines, chitosan-coated magnetic NPs (CS-MNPs) enhance drug accumulation by 63% and cause a 54% increase in apoptosis, according to Taherian et al. (2021) [46]. In their study on colorectal cancer, Farmanbar et al. (2022) showed that CS-MNPs were effective in delivering oxaliplatin (82% EE) and irinotecan (87% EE), with sustained release rates of 68% and 72% over 48 h, respectively, in settings resembling tumors. Research also shows that the bioadhesiveness of chitosan and magnetic targeting work together to reduce the viability of cancer cells by 62% and increase the drug’s half-life by 2.3 times. These results show that NPs made of chitosan are safer and more effective than traditional chemotherapy because they improve drug solubility, focused cytotoxicity, and prolonged release [47].

### 2.2. Ocular and Nasal Drug Delivery

Because of its special mucoadhesive qualities, chitosan is an excellent material to use in medication administration systems for the eyes and nose. Because of its mucosal adhesive properties, the biopolymer increases the retention period of therapeutic drugs at the site of action, which is critical for better medication absorption and therapeutic benefits. Because localized distribution may lessen systemic adverse effects and enhance therapeutic effectiveness, this characteristic is especially important in applications linked to the eye and nose passages [48,49,50].

Chitosan NPs have shown to be far more effective than traditional techniques of medication delivery when it comes to ocular administration. Silva et al. (2017) found that compared to conventional eye drops or other formulations, chitosan-based NPs may enhance ocular medication delivery effectiveness by a factor of three [51]. Because chitosan has such strong mucoadhesion, the formulation stays in touch with the eye surface for a longer period of time, increasing its efficacy. Particularly helpful for long-term eye conditions including glaucoma and dry eye syndrome, this results in reduced dosage requirements, improved patient compliance, and far less frequent administration [52,53,54,55].

**Figure 2:**
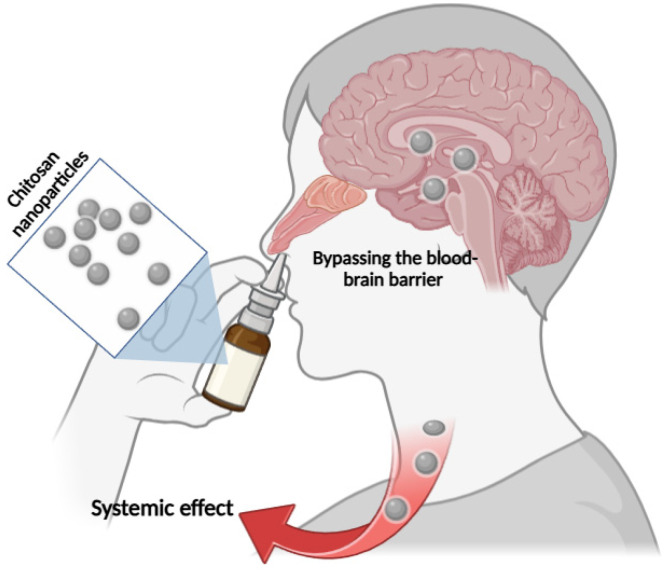
The cerebral and systemic effects of nasally administered chitosan NPs.

Nasal drug delivery using chitosan NPs circumvents the drawbacks of conventional systemic administration methods, which frequently result in a low bioavailability of therapeutic agents aimed at the central nervous system (CNS). Bypassing the blood–brain barrier (BBB), a crucial obstacle in drug development, is possible via the nasal route, allowing for direct access to the circulation (Figure 2) [50,56,57].

Research by Seyam et al. (2020) indicates that hydrophilic biopharmaceuticals, such as insulin, may be more efficiently transported via the nasal passages when encased in chitosan NPs. Their research showed that these NPs might outperform traditional delivery techniques in terms of bioavailability, reaching a rate of around 30%. Because of chitosan’s capacity to temporarily open tight connections between epithelial cells and mucoadhesion’s effect on increasing contact time with the nasal epithelium, this improvement has been achieved [58].

An ideal carrier for nasal medication administration, especially for systemic and nose-to-brain transfer, chitosan may momentarily open tight connections in the nasal epithelium. Drug bioavailability is 2.5 times higher in formulations including chitosan compared to those without, according to research by Kaur et al. (2023), because chitosan increases retention time, which in turn improves drug absorption [59]. Quantitative data on domperidone-loaded chitosan–ethyl cellulose microspheres were presented by Zafar et al. (2021). These microspheres showed a 1.9-fold increase in systemic bioavailability compared to oral administration and a 68% drug release within the first 6 h. This indicated that chitosan could evade first-pass metabolism [60]. Subsequently, Noorulla et al. (2022) show a 3.2-fold increase in drug concentration in brain tissue compared to oral treatment and a 72% drug release over 12 h using intranasal Buspirone-loaded chitosan-decorated nanostructured lipid carriers [61].

In addition, Viswanadhan Vasantha et al. (2023) look at in situ nasal gels that carry chlorpheniramine and loratadine by mixing chitosan with cellulose derivatives. Their research demonstrates that the medicine is released gradually over the course of 24 h, which means that the antihistaminic benefits last longer and that less frequent dosage is required [62]. The study conducted by Georgieva et al. (2023) examines the feasibility of using chitosan-based NPs to deliver galantamine intranasally for the treatment of Alzheimer’s disease. The researchers discovered that the drug was taken in 2.7 times more effectively by the nasal epithelium and that there was a marked improvement in brain acetylcholine levels, indicating better drug targeting [63]. In a follow-up study, Ways et al. (2022) looked at mucus-penetrating chitosan NPs that had been grafted with non-ionic polymers. They found that the drug absorption efficiency was improved, and the diffusion rate through nasal mucus was 40% quicker than unmodified chitosan [64]. Taken together, these results suggest that chitosan is a great polymer for nasal drug administration, with the potential to increase targeting, retention time, and permeability—all of which are important for treatments involving the central nervous system.

The bioadhesiveness, increased corneal permeability, and sustained drug release capabilities of chitosan-based delivery systems make them ideal for ocular drug administration, which in turn reduces the frequency of dosage. Analyzing olopatadine-loaded chitosan NPs for the treatment of allergic conjunctivitis, Güven and Başaran (2021) reported a drug EE of 81%, which greatly improved the stability of the formulation. The researchers also discovered that chitosan NPs improve therapeutic efficiency with less dosage frequency than traditional eye drops by increasing corneal penetration by a factor of three. This, in turn, causes a prolonged release of the medicine for up to twelve hours. Because of these characteristics, chitosan is an excellent carrier for ophthalmic medications that need to stay on the eye’s surface for a long time [65].

Further investigation into chitosan-modified gellan gum hydrogels for the sustained release of timolol maleate in glaucoma treatment is carried out by Shajari et al. (2024). Their results show that the hydrogel formulation successfully prolongs the lowering of intraocular pressure (IOP) for at least another 24 h, with a sustained medication release of 76% compared to 40% for hydrogels that do not include chitosan. Because of the need of maintaining a constant IOP during glaucoma therapy, this regulated release profile is of the utmost importance [66]. Because chitosan improves ocular surface adhesion and permeability, stimuli-responsive ocular drug carriers based on chitosan were investigated by Berillo et al. (2021), who found that the medication’s bioavailability was increased by 2.8 times. They also found that the cornea is negatively charged; therefore, the positive charge of chitosan improves electrostatic interactions, which increases medication retention [67]. These characteristics, together with chitosan’s minimal toxicity and biocompatibility, make it an ideal polymer for ocular medication administration, which guarantees longer-lasting therapeutic benefits, better drug absorption, and more patient compliance.

### 2.3. Pulmonary Delivery

As a new method for pulmonary medication administration, chitosan NPs serve as efficient carriers that allow therapeutic substances to be directed to the lungs. The enormous surface area and extensive circulatory network of lung tissues provide a unique potential for targeted medication administration with low systemic effect, making this application especially crucial. The increased bioavailability is mainly due to the fact that chitosan NPs have better permeability through the lung epithelial lining. This is because their small size and optimized surface characteristics make them less likely to be cleared by mucociliary action [68,69,70,71,72].

When it comes to chitosan NPs’ deposition in the pulmonary system, particle size and shape are crucial variables. Optimal deposition in alveolar areas is achieved by NPs with sizes between 200 and 300 nm, according to research. In order to improve treatment results for a variety of respiratory illnesses, this size range is optimal for penetrating deeply into the lungs and overcoming the clearance mechanisms of the respiratory tract [7,73].

When it comes to treating lung disorders like TB, the capacity to create targeted medication concentrations is very crucial. Long treatment periods and large medication dosages are common in tuberculosis therapy, which increases the risk of serious systemic adverse effects. It has been suggested that chitosan NPs might be used to guide medication delivery to the site of infection, which could lead to a decrease in drug dose and an improvement in the therapeutic index [74,75,76,77].

Chitosan NPs may be engineered to break down in the pulmonary environment, releasing their therapeutic payload in a regulated way thanks to their natural biodegradable qualities. This characteristic improves the treatment’s efficacy and safety by reducing the likelihood of adverse effects caused by drug accumulation in the tissues.

By creating chitosan extra-fine particles coated with cyclosporine A, Huang et al. (2023) surpassed traditional formulations and demonstrated exceptional deep lung penetration with an 87% fine particle fraction. To ensure extended immunosuppressive effects and reduce systemic toxicity, their in vivo pharmacokinetic investigations showed a 3.2-fold increase in lung drug retention compared to traditional inhalation treatment. Moreover, their study of inflammatory markers showed that treated mice had a 68% decrease in IL-6 and TNF-α levels, which validates the function of chitosan in reducing inflammation in the lungs [78]. Peng et al. (2021) enhanced the aerodynamic drug deposition by 75% and achieved an improved mass median aerodynamic diameter of 2.3 µm, guaranteeing efficient alveolar targeting, by utilizing supercritical CO₂-assisted preparation of chitosan-based nano-in-microparticles [79]. This provides further evidence that the formulation features of chitosan greatly improve medication distribution in the lower airways.

Using chitosan-based nanocarriers for targeted delivery, Kazmi et al. (2023) were able to treat lung cancer. They achieved a 2.8-fold increase in cellular drug absorption, which translated to a 65% decrease in tumor growth after four weeks [80]. The findings are in line with those of Yan et al. (2021), who created a microgel of carboxymethyl chitosan and hyaluronic acid that was coordinated with zinc ions. The microgel demonstrated an 85% sustained drug release over 24 h, which prevented the medication from being cleared quickly and increased the therapeutic lifespan [81]. The antiviral potential of chitosan was further investigated by Tousian and Khosravi (2023). They showed that pulmonary vaccine carriers based on chitosan elicited a mucosal immune response that was 78% stronger than that of traditional inhaled vaccines. This finding raises the possibility of using chitosan in immunotherapies against respiratory viruses like COVID-19 [82]. Finding that N,N,N-trimethyl chitosan increases medication permeability and absorption across alveolar membranes by 2.5-fold, Szabová et al. (2023) provided a mechanistic insight into the process [83]. The results are summarized in a study by Alwahsh et al. (2024), who drew the conclusion that pulmonary drug delivery systems based on chitosan increase therapeutic effectiveness and patient adherence by reducing dose frequency by 50% and prolonging drug half-life by three to four times [84].

Taken together, these studies demonstrate that chitosan may enhance the targeting of pulmonary drugs, increase their bioavailability, and guarantee their sustained release for a wide range of respiratory uses, including immunosuppressive, lung cancer therapy, antiviral, and chronic illness management.

### 2.4. Gene Delivery

As an effective non-viral alternative to viral transfection, chitosan NPs have shown great promise as gene delivery vehicles. Stable complexes may be formed via electrostatic interactions between positively charged chitosan and negatively charged nucleic acids. Important for successful gene therapy, this trait greatly improves cellular absorption of genetic material [19,85].

Research has demonstrated that several chitosan combinations increase doxorubicin concentrations in targeted areas, demonstrating chitosan’s promise as an adaptable medication delivery system. Importantly, chitosan may improve the absorption of connected nucleic acids by cells, leading to remarkable transfection rates of more than 80% in several cell lines. The remarkable efficacy of chitosan nanosystems in delivering genetic payloads across biological barriers is a game-changer in the fight against cancer and other genetic illnesses [86,87,88,89,90].

By including targeting ligands or other biopolymer systems, gene delivery may be made more selective and much more effective. For example, research conducted by Nam et al. (2009) found that glycol chitosan NPs that had been hydrophobically modified had better cellular absorption than their non-modified equivalents. Research is now focused on improving the effectiveness of chitosan NPs as gene carriers by structural and functional modifications [91].

### 2.5. Systemic Drug Delivery

Particularly for hydrophilic medicines, chitosan NPs have the potential to increase their oral bioavailability and hence improve systemic drug delivery. When cyclosporine A was synthesized as chitosan NPs for oral delivery, the experiments carried out by Kean et al. (2010) showed that its systemic bioavailability increased significantly, reaching up to 60%. The protective action of NPs against enzymatic degradation in the gastrointestinal system is mainly responsible for these increases in bioavailability [92].

Because of its mucoadhesive qualities, chitosan plays a crucial role in increasing the bioavailability of drug-loaded NPs by enhancing their retention at absorption sites. With its ability to improve NP–intestinal mucosa contact, chitosan affects the kinetics of medication absorption and the overall therapeutic effectiveness of orally delivered medicines. Medications that need to be taken at regular therapeutic levels in order to work at their best must have the capacity to maintain drug release and aid absorption [15,93,94,95,96].

A number of factors, such as the amount of deacetylation and the molecular weight of chitosan, may be adjusted to fine-tune the release profile of chitosan NPs. Scientists may use these modifiable elements to create medications with tailored delivery systems that react to certain physiological signals or release pharmaceuticals over long durations [14,97,98].

Therapeutic compounds with low bioavailability may be more easily absorbed when encased in chitosan NPs, which can cross the intestinal barrier. Because of this promise, chitosan NPs are becoming more important in the field of systemic treatment, especially for hydrophilic medicines that have trouble being absorbed.

### 2.6. Physiotherapy and Bone Regeneration

**Figure 3:**
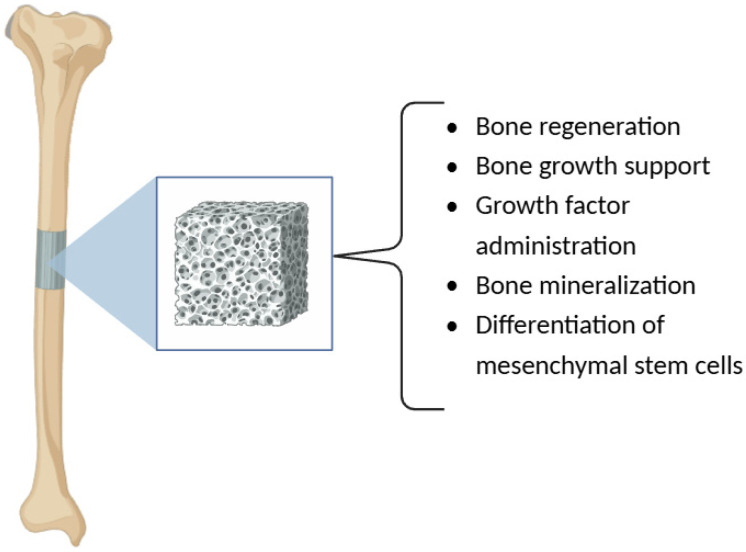
The effects of chitosan scaffolds on bone regeneration.

Based on the obtained results, it can be concluded that chitosan nanofibers, when incorporated into various composite systems, act as an effective substrate to enhance the osteogenic activity. Tao et al. (2020) showed that chitosan nanofibers support cell attachment and proliferation, both of which are essential for bone regeneration (Figure 3) [99]. Additionally, combining chitosan with growth factors and morphogenetic proteins enhances the regenerative capability of the scaffolds and presents a prospective solution for the potential restoration of damaged skeletal systems, these chitosan scaffolds promote the biological response, which is necessary for the regeneration of new bone [100,101,102,103].

This, however, is not new, and previous research has shown that the incorporation of chitosan into scaffold design improves biological interactions due to the bioactive properties of the material. For example, Fasolino et al. (2019) found that chitosan-based scaffolds are osteoinductive and anti-inflammatory and are thus useful in modifying the body’s response to healing [104]. The effect of chitosan/gelatin scaffolds osteoblasts has been found necessary for bone mineralization support and the differentiation of mesenchymal stem cells [105].

By comparing chitosan–calcium phosphate scaffolds to untreated defects in animal models, Liu et al. (2022) show that chitosan–calcium phosphate scaffolds increase bone mineral density by 46%. This highlights the function of chitosan in mineralized composite scaffolds [106]. Another study that supports quicker bone regeneration found that chitosan–hydroxyapatite composites increased osteoblast proliferation by 72% [107]. Analyzing chitosan-based scaffolds loaded with growth factors, Bharathi et al. (2022) found that their bioactive potential was shown by a 3.1-fold increase in alkaline phosphatase activity, a critical hallmark of osteoblast development [108]. In addition, Ji et al. (2022) created injectable hydrogels containing hydroxypropyl chitin and porous chitosan microspheres for use in osteochondral repair. These hydrogels allowed for a 21-day sustained drug release and a 2.4-fold increase in chondrogenic gene expression, suggesting better regeneration of the cartilage–bone interface [109].

Osteomyelitis and osteoporosis are two conditions that show promise when treated with drug delivery systems derived from chitosan. In their study, Tao et al. (2021) created chitosan microspheres loaded with antibiotics. These microspheres were able to eradicate 92% of *Staphylococcus aureus* germs, which greatly decreased bone loss caused by infections [110]. In their study, Gao et al. (2023) showcased an improved method using a chitosan–vancomycin hydrogel scaffold. They used a dual-controlled drug release mechanism that kept therapeutic concentrations stable for more than 28 days. This allowed for sustained antibacterial action and supported bone healing simultaneously [111]. By combining photothermally induced chitosan-reduced graphene oxide hydrogel with teriparatide administration, Wang et al. (2021) created a solution for osteoporosis that increased new bone production by 61%. This was achieved by a combination of prolonged drug release and localized heat stimulation [112].

Chitosan is an incredibly useful biomaterial for orthopedic applications because of its controlled drug release, osteoconductivity, and antibacterial activity, all of which contribute to its flexibility in bone healing.

## 3. Stability and Characterization of Chitosan NPs

**Figure 4:**
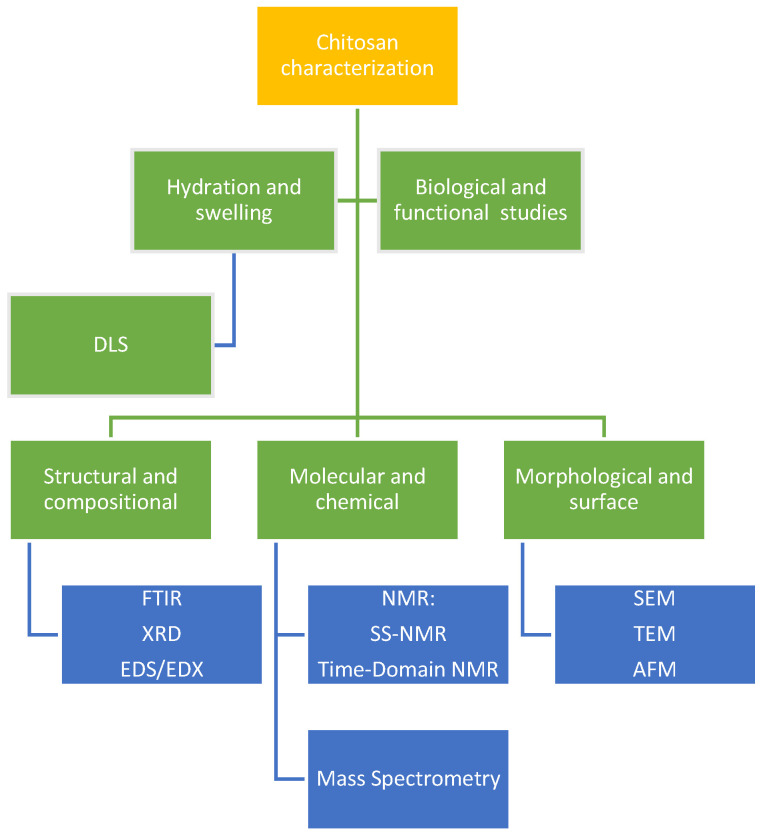
Characterization methods for chitosan nanostructures.

In order to use chitosan in medication delivery and biomaterials, its structural properties must be determined. Many studies have used solid-state nuclear magnetic resonance (SSNMR) to examine the molecular dynamics, crystallinity, and polymer interactions of chitosan (Figure 4). Wang et al. (2021) found that film flexibility and drug release kinetics are correlated with anisotropic motion and different microdomain structures [113]. By using principal component analysis on SSNMR data, Fernando et al. (2021) established a connection between the degree of deacetylation and polymer flexibility and mucoadhesion [114]. In contrast, Facchinatto et al. (2021) utilized time-domain NMR relaxometry to shed light on the water-binding capacity and hydration states that impact bioavailability [115].

Additional information on functional changes and crystallinity may be gleaned by X-ray diffraction (XRD) and Fourier transform infrared spectroscopy (FTIR). Important amide and hydroxyl stretching bands were identified by Zhou et al. (2021), who proved that the enzymatic depolymerization was effective in improving solubility [116]. Asl et al. (2023) [117] showed that metal–organic framework (MOF) integration shifted chitosan to an amorphous form, improved drug loading and regulated release characteristics, while Kazemi et al. (2021) employed FTIR to validate chitosan–magnetite–GO hybrid functionalization. Improved porosity and medication diffusion rates were correlated with reduced crystallinity, as proven by XRD measurements, in modified chitosan composites [118].

NPs of a size less than 300 nm are usually the most effective for increasing cellular absorption, according to DLS tests. In addition to being less likely to be cleared by the immune system, particles of this size may infiltrate cellular membranes more easily. NPs’ surface morphology affects their interactions with biological systems, and SEM gives extensive insights into this morphology. By examining these features, scientists might learn how NPs’ physical qualities could be transformed into their biological efficacy [119,120,121].

Additional levels of information, notably regarding the thermal stability of chitosan NPs, may be gained from studies that use differential scanning calorimetry (DSC). Scientists may learn about the physicochemical stability of NPs under physiological settings by measuring their temperature responses; this knowledge informs the trustworthiness of therapeutic formulations [122,123,124].

To guarantee viability and repeatability in clinical applications, it is crucial to understand and manage parameters including size distribution, shape, and stability. Not only is chitosan NPs’ stability important while they are stored, but it is also critical when they are moving through the circulation and when they reach their target. Reduced effectiveness or increased toxicity may result from changes in the drug delivery profile caused by particle aggregation, degradation, or changes in surface characteristics [125,126,127].

One important element of characterizing chitosan NPs is testing their biodistribution and pharmacokinetics in living organisms. The potential for therapeutically useful uses of these NPs is dependent on our ability to understand their behavior in a biological system, including their absorption, distribution, metabolism, and excretion. Researchers have developed techniques like radiolabeling and fluorescence imaging to trace the path of administered NPs, allowing them to learn more about how the properties of the particles affect the therapeutic effects they produce [128,129,130].

For a formulation to last, thermal stability, as measured by TGA and DSC, is crucial. In order to guarantee stability under physiological settings, Manjusha et al. (2023) found that chitosan–polymer networks degraded in two stages, from 100 to 320 °C [131]. The incorporation of MOF/GO hybrids increased decomposition temperatures (ΔT~35 °C), which improved the lifespan of the implant and allowed for regulated drug release, according to Asl et al. (2023). The morphology, porosity, and bioadhesion of the nanostructure were studied using scanning electron microscopy (SEM), transmission electron microscopy (TEM), and atomic force microscopy (AFM) [117]. Sarma et al. (2022) confirmed the uniform dispersion of the nanoparticles and the formation of spherical core–shell structures, which are important for improved cellular uptake and long-term drug delivery [132].

By combining high-resolution microscopy, thermal analysis, FTIR, XRD, and SSNMR, the drug carrier potential of chitosan has been enhanced by optimization of bioadhesion, controlled release, and structural stability. To further boost chitosan’s biomedical uses in targeted therapeutics and regenerative medicine, future studies should combine real-time in situ analytical methods including XPS, Raman mapping, and dynamic light scattering.

### 3.1. Chitosan—The Superior Biopolymer

Biodegradability, biocompatibility, and the capacity to generate stable, pH-sensitive nanoparticles are just a few of the distinctive properties that have repeatedly positioned chitosan as an ideal biopolymer for use in a range of drug delivery systems. Chitosan outperformed other biopolymers in terms of controlled release profiles and drug encapsulation efficiency in a number of trials. Desai et al. (2023) found that the encapsulation efficiency of chitosan nanoparticles was over 90%, while systems based on gelatin and alginate only achieved 70–80% [133]. Similarly, compared to alginate (65% release) and collagen (55% release) during the same 72 h period, chitosan nanocarriers demonstrated an 85% drug release [134]. In addition, Omer et al. (2021) found that at pH 1.2 after 4 h, a pH-sensitive chitosan–gelatin hydrogel released 80% of the medication, compared to 55% for gelatin alone [135]. The data presented here suggest that chitosan is the superior option for uses requiring sustained therapeutic effects because of its more efficient and longer drug release.

Chitosan is remarkable for more than only its encapsulation and release efficiency; it also stands out for its unique properties, such as its appropriateness for oral drug administration and its capacity to increase bioavailability via mucoadhesion. For example, Syed et al. (2023) highlighted that chitosan may enhance the solubility and stability of medications in the gastrointestinal system because of its cationic character, which enables it to interact efficiently with negatively charged biomolecules [136]. Showing that chitosan has better control over release kinetics, Fazal et al. (2023) found that chitosan–gelatin composites produced a 75% drug release after 48 h, compared to just 55% for gelatin alone [137]. Hisham et al. (2024) also discovered that chitosan-based scaffolds expedited wound closure in wound healing applications, with full healing accomplished in 10 days as opposed to 14–16 days for non-chitosan scaffolds [138].

Supported by statistical proof and real-world performance, these findings highlight chitosan’s specific advantages in numerous biomedical applications, establishing it as a top contender for drug delivery and therapeutic uses.

### 3.2. Challenges and Limitations

There are a number of obstacles and restrictions that must be overcome before chitosan NP drug delivery systems may reach broad clinical use, despite their promising characteristics and many potential uses. A big problem is that different materials might have different molecular weights and degrees of deacetylation, which can cause important characteristics to be inconsistent. The therapeutic effectiveness of chitosan NPs might be affected by these variances, as materials with varied biological characteristics are often produced by various sources. It is crucial to design a thorough methodology for standardizing the extraction and processing of chitosan in order to obtain uniform quality across batches [139,140,141].

The use of chitosan NPs in therapeutic settings is complicated and fraught with regulatory red tape. Comprehensive evaluations of their biodistribution, biodegradation processes, and possible long-term toxicity are required prior to their usage in therapeutic contexts. In order to resolve any safety issues related to chitosan nanocarriers, these studies are essential. This is especially true when it comes to how these nanocarriers interact with biological systems. The possibility of immunogenicity linked to chitosan NPs should also not be disregarded. Despite chitosan’s biocompatibility, there are differences in residual extraction and purity. Some people’s immune systems may react negatively to solvents or other manufacturing-related substances. Preserving patients’ well-being requires research into the immunological profiles and potential long-term impacts of these NPs [142,143].

Addressing the issues of variability, scalability, regulatory compliance, and safety is crucial for the effective deployment of chitosan NPs as drug delivery systems in clinical practice, despite their tremendous promise. To fully use chitosan NPs in therapeutic applications, further study into these areas is necessary. This will lead to improved health outcomes via more sophisticated drug delivery methods.

## 4. Future Research Directions

Customizing medicines for individual patients is the future of chitosan-based drug delivery systems. This might greatly improve the therapeutic potential and decrease negative effects. An exciting new direction in cancer research is the use of patient-derived tumor organoids and 3D cell culture models to create formulations tailored to each patient’s specific molecular profile. These models allow for the evaluation of chitosan NP formulations within the setting of the patient’s tumor microenvironment, and they also more closely recreate tumor heterogeneity than typical 2D cultures. Important insights into particular biomarkers for targeted medication administration may be gained by integrating chitosan NP systems with multi-omics data, which includes genomic, transcriptomic, and proteomic profiles. It is possible to create customized formulations for the targeted delivery of chemotherapeutics, RNA-based treatments (e.g., siRNA, mRNA), and immunomodulatory drugs (e.g., immune checkpoint inhibitors). Combination medicines that are given using chitosan-based platforms have the potential to enhance immune system activation and overcome cancer cells’ resistance mechanisms through synergistic interactions. The potential for chitosan NPs to be used in next-generation immunochemotherapy is enhanced by the use of immune-modulatory drugs like anti-CTLA-4 or anti-PD-1/PD-L1 antibodies, which, in conjunction with cytotoxic therapies, might stimulate the immune system to fight tumors.

The chemical modification and functionalization of chitosan to increase its pharmacological characteristics will be a major area of study in the quest for better chitosan-based formulations. If chitosan were to undergo surface property modifications such as N-acylation or PEGylation, its solubility, biocompatibility, and blood circulation time might all be enhanced. To counteract the fast excretion of nanoparticles from the bloodstream, this is crucial. We should look at hybrid NPs that combine chitosan with other biodegradable or synthetic polymers like PLGA (poly(lactic-co-glycolic acid)), hyaluronic acid, or polylactic acid (PLA) to improve stability and controlled drug release. Some potential benefits of these hybrid NPs include less toxicity, better tissue penetration, and prolonged, sustained therapeutic release. In addition, small-molecule ligands, monoclonal antibodies, or aptamers may be used to functionalize chitosan NPs. These ligands bind selectively to tumor cell receptors that are overexpressed, such as HER2, EGFR, or folate receptors. Treatment efficacy and safety would be greatly improved with the use of such targeted tactics, which would increase selectivity while decreasing off-target effects. To optimize these tailored systems, research into endocytosis routes and ligand-receptor binding kinetics is crucial.

It will be critical to make more progress in nanoparticle characterization and biological interactions, in addition to developing and improving chitosan NPs. Before chitosan formulations are used in clinical trials, more relevant preclinical testing platforms for evaluating their therapeutic potential and safety profiles might be patient-derived xenografts (PDX) or organ-on-a-chip, two examples of ex vivo models. In order to track the biodistribution, pharmacokinetics, and cellular destiny of chitosan NPs in real time, it is recommended to include modern imaging methods such as positron emission tomography (PET), fluorescence lifetime imaging (FLI), and magnetic resonance imaging (MRI) into in vivo studies.

Imaging methods that combine MRI and PET, known as multimodal imaging, might help scientists learn more about tissue targeting, clearance routes, and any off-target effects. By modeling interactions between nanoparticles and biological components, computational models including machine learning methods might be used to forecast how chitosan NPs would behave inside biological systems, providing a quicker path to clinical translation.

## 5. Conclusions

NPs made of chitosan are a huge step forward in the delivery of drugs; they provide a flexible platform that has several uses in cancer treatment, gene therapy, and pulmonary administration, among many other therapeutic areas. Their capacity to create stable, mucoadhesive NPs, their biodegradability, and their biocompatibility are some of their unique physicochemical qualities that make them ideal for improving the solubility and bioavailability of drugs.

There have been notable advancements in the delivery of drugs via nasal, ocular, and pulmonary applications using nanocarriers derived from chitosan. When compared to more conventional means of medication administration, research shows that chitosan nanoparticles may increase the bioavailability of hydrophilic medicines, such as insulin, by as much as 30% when administered nasally. The capacity of chitosan to momentarily loosen tight connections in the nasal epithelium is responsible for this enhancement, which in turn results in enhanced drug absorption and prolonged retention periods. Chronic therapies may benefit from the prolonged release profiles offered by chitosan nanoparticles because of their capacity to circumvent first-pass metabolism and their ability to enhance drug bioavailability in systemic distribution by a factor of 2.5 compared to non-chitosan formulations.

The corneal permeability and drug stability of chitosan-based formulations for ocular medication administration are both enhanced. For example, research shows that chitosan nanoparticles tripling the bioavailability of eye medication improve therapeutic effectiveness and patient compliance by decreasing dosage frequency. Nanoparticles of chitosan have also shown enhanced deposition in the alveolar areas during pulmonary drug administration; particles ranging in size from 200 to 300 nm were found to have the best lung penetration. Direct distribution to the lungs may decrease systemic adverse effects and improve treatment results, making this targeting capacity especially relevant for respiratory disorders like TB and lung cancer.

Because of its biodegradability, mucoadhesion, and capacity to improve medication retention, chitosan is a promising material for a number of medicinal uses. When coupled with materials such as calcium phosphate and hydroxyapatite, chitosan scaffolds have shown an increase in bone mineral density of 46% and an increase in osteoblast proliferation of 72% during bone regeneration. Another important step in osteoblast formation, alkaline phosphatase activity, was shown to be 3.1 times higher in chitosan nanoparticles that were loaded with growth nutrients.

These results demonstrate chitosan’s adaptability in regenerative medicine and drug delivery; however, there further studies must be conducted to solve problems with nanoparticle stability and optimize drug release for clinical uses.
